# Supplementary material for: Identification of photoperiod-induced specific miRNAs in the adrenal glands of Sunite sheep (Ovis aries)
Source: Front Vet Sci. 2022 Jul 22;9:888207. doi: 10.3389/fvets.2022.888207 (PMC9354845; doi:10.3389/fvets.2022.888207)
Supplement: Supplementary file 1 [file Data_Sheet_1.ZIP › Supplementary file/miRDeep2 file.zip!/miRDeep2 file/Know_Structure/SPLP42A2/oar-mir-3959.pdf]

miRBase precursor : oar-mir-3959
Total read count : 20723
oar-miR-3959-5p read count : 18599
oar-miR-3959-3p read count : 2123
remaining reads : 1

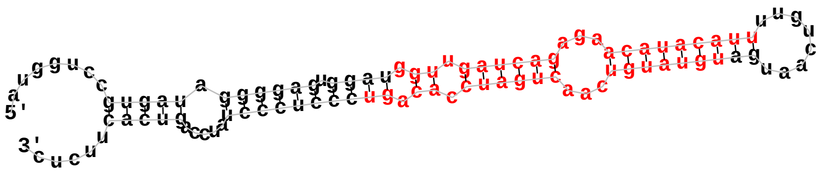

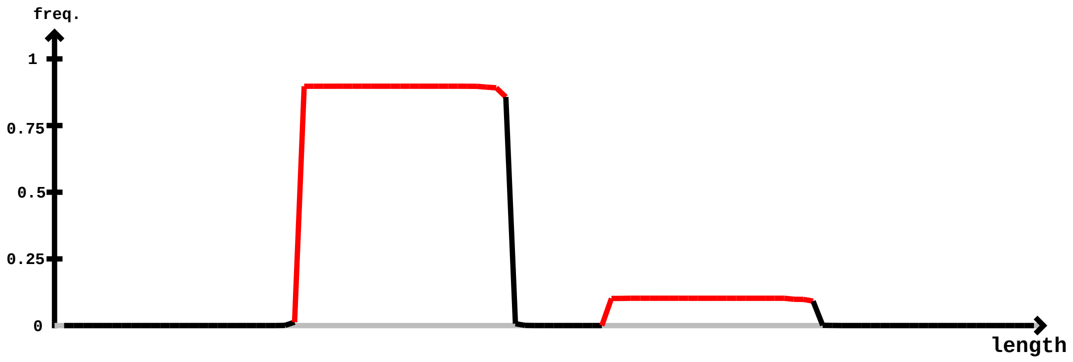

oar-miR-3959-5p

oar-miR-3959-3p

```
5'- augguccgugauaggggaguggaugguugaucagagaacauacauuuugucaaugauguaugucaacugauccacagucccucccuauccagucacuucuc   -3'   exp
    .......(((((.((((((.((((.((.((((((...(((((((((........)))))))))...)))))).)).))))))))))).....))))).....   reads   mm   sample
    ......................augguugaucagagaacau............................................................   2       0    seq
    ......................augguugaucagagaacaua...........................................................   2       0    seq
    ......................augguugaucagagaacauac..........................................................   5       0    seq
    ......................augguugaucagagaacauaca.........................................................   3       0    seq
    .......................ugguugaucagagaacauac..........................................................   4       0    seq
    .......................ugguugaucagagaacauaca.........................................................   5       0    seq
    .......................ugguugaucagagaacauacau........................................................   13      0    seq
    .......................ugguugaucagagaacauacauu.......................................................   228     0    seq
    .......................ugguugaucagagaacauacauuu......................................................   1       0    seq
    ........................gguugaucagagaacau............................................................   2       0    seq
    ........................ggGugaucagagaacau............................................................   1       1    seq
    ........................gguugaucagagaacaua...........................................................   4       0    seq
    ........................gguugaucagagaacauac..........................................................   54      0    seq
    ........................gguugaucagagaacauaca.........................................................   43      0    seq
    ........................gguugaucagagaacauacau........................................................   699     0    seq
    ........................gguugaucagagaacauacauu.......................................................   17386   0    seq
    ........................gguugaucagagaacauacauuu......................................................   131     0    seq
    ........................gguugaucagagaacauacauuuu.....................................................   10      0    seq
    ........................gguugaucagagaacauacauuuugu...................................................   2       0    seq
    .........................guugaucagagaacauacauu.......................................................   2       0    seq
    ...........................ugaucagagaacauacauu.......................................................   1       0    seq
    ...........................ugaucagagaacauacauuuu.....................................................   1       0    seq
    ...........................................auuuugucaaugauguauguc.....................................   1       0    seq
    ........................................................uguaugucaacugaucca...........................   1       0    seq
    ........................................................uguaugucaacugauccac..........................   78      0    seq
    ........................................................uguaugucaacugauccaca.........................   11      0    seq
    ........................................................uguaugucaacugauccacag........................   133     0    seq
    ........................................................uguaugucaacugauccacagu.......................   1880    0    seq
    ........................................................uguaugucaacugauccacaguc......................   4       0    seq
    .........................................................guaugucaacugauccacagu.......................   1       0    seq
    ..........................................................uaugucaacugauccacaguc......................   6       0    seq
    ..........................................................uaugucaacugauccacagucc.....................   9       0    seq
```
